# Chemotactic Responses of *Xanthomonas* with Different Host Ranges

**DOI:** 10.3390/microorganisms11010043

**Published:** 2022-12-22

**Authors:** Marta Sena-Vélez, Elisa Ferragud, Cristina Redondo, James H. Graham, Jaime Cubero

**Affiliations:** 1 Laboratoire de Biologie des Ligneux et des Grandes Cultures (LBLGC) EA 1207, L’institut National de Recherche pour L’agriculture, L’alimentation et L’environneme (INRAE) USC1328, Orléans University, BP 6759, CEDEX 2, 45067 Orléans, France; 2 Instituto Nacional de Investigación y Tecnología Agraria y Alimentaria (INIA/CSIC), 28040 Madrid, Spain; 3 Citrus Research and Education Center (CREC), University of Florida, 700 Experiment Station Road, Lake Alfred, FL 33850-2299, USA

**Keywords:** *Citrus*, xanthomonads, bacterial motility, MCPs, methyl-accepting chemotaxis proteins

## Abstract

*Xanthomonas citri* pv. *citri* (*Xcc*) (*X. citri* subsp. *citri*) type A is the causal agent of citrus bacterial canker (CBC) on most *Citrus* spp. and close relatives. Two narrow-host-range strains of *Xcc*, A^w^ and A*, from Florida and Southwest Asia, respectively, infect only Mexican lime (*Citrus aurantifolia*) and alemow (*C. macrophylla*). In the initial stage of infection, these xanthomonads enter via stomata to reach the apoplast. Herein, we investigated the differences in chemotactic responses for wide and narrow-host-range strains of *Xcc* A, *X. euvesicatoria* pv. *citrumelonis* (*X. alfalfae* subsp. *citrumelonis*), the causal agent of citrus bacterial spot, and *X. campestris* pv. *campestris*, the crucifer black rot pathogen. These strains of *Xanthomonas* were compared for carbon source use, the chemotactic responses toward carbon compounds, chemotaxis sensor content, and responses to apoplastic fluids from *Citrus* spp. and Chinese cabbage (*Brassica pekinensis*). Different chemotactic responses occurred for carbon sources and apoplastic fluids, depending on the *Xanthomonas* strain and the host plant from which the apoplastic fluid was derived. Differential chemotactic responses to carbon sources and citrus apoplasts suggest that these *Xanthomonas* strains sense host-specific signals that facilitate their location and entry of stomatal openings or wounds.

## 1. Introduction

Citrus bacterial canker (CBC) is one of the most important bacterial diseases of citrus in the tropical and subtropical areas of the world. CBC is characterized by the appearance of necrotic, erumpent lesions on leaves, fruits, and stems and may cause premature defoliation and fruit drop in most *Citrus* species and close citrus relatives in the family *Rutaceae* [1,2,3]. Distinct types of CBC have been described caused by different bacteria within the genus *Xanthomonas*. The symptoms of these canker diseases are similar, and initially, all the causal bacterial strains were classified within the same species of the genus [1,2,4,5,6,7]. The most studied and widespread CBC type is the Asiatic citrus canker or type A, which comprises two pathotypes, A* and A^w^, that have been characterized as genetically slightly distinct from the *Xanthomonas citri* pv. *citri* (*Xcc*) type A [8,9,10,11]. A* and A^w^ occur in Southwest Asia and Florida, respectively, and have narrow host ranges that include Mexican lime (*C. aurantifolia*) and alemow (*C. macrophylla*). Although in the field, these strains only cause disease on lime, when they are infiltrated into the leaves of other citrus species, they produce atypical lesions, slightly raised with no rupture of the epidermis [8,9]. Furthermore, the *Xcc* A^w^ strain is able to cause a hypersensitive response on Duncan grapefruit (*C. paradisi*) when infiltrated [8].

Chemotaxis is the mechanism enabling bacteria to sense stimuli, such as nutrients, light, or temperature, that attract them to the site that is optimally suited for host colonization and infection [12]. Several studies on plant pathogens have demonstrated the importance of this mechanism; for example, jasmonate is a plant signal that attracts *Dickeya dadantii* to wounds, facilitating entry of the host and enhancing the infection process [13,14]. Chemotaxis- and motility-related genes were overexpressed during the epiphytic stage of the interaction on bean leaves with *Pseudomonas syringae* but not after reaching the apoplast [15]. In *X. campestris* pv. *campestris* (*Xc*), *cheY* and XAC0324 genes have been associated with chemotaxis in host leaf colonization, although once the bacteria reach the apoplastic space, the participation of chemotaxis is unimportant for symptom development in cabbage [16].

Methyl-accepting chemotaxis proteins (MCPs) are protein receptors present on both, the bacterial membrane and the cytoplasm, able to sense environmental clues and trigger a motile response to favor bacterial fitness and survival in the environment [17,18]. Diverse MCPs were identified in *Xanthomonas* spp., including *Xcc* [19,20].

The role of chemotaxis in the *Xcc* infection progress in Duncan grapefruit has been suggested [21], and the requirement for active bacterial motility and chemotaxis on the plant surface to locate and specifically colonize the host apoplastic site is supported also by indirect evidence. *Xcc* is dispersed by wind and rain; on the leaf surface, *Xcc* is able to swim short distances reaching the plant interior through stomata or wounds. This process is facilitated by the action of wind but also happens in its complete absence [22,23,24]. If there is water on the leaf surface, bacterial movement and entry into stomata or wounds may be mediated by chemotaxis. This hypothesis was reinforced by confocal laser scanning microscopy visualization of *Xcc* A on citrus, which showed bacterial accumulation at the edge of the stomata immediately after the spray-inoculation of leaves [25]. Furthermore, wide-host-range *Xcc* and *X. euvesicatoria* pv. *citrumelonis* (*Xec*) were detected in the apoplast of Swingle citrumelo leaves, while the non-host strain *Xcc* A^w^ type was not present. In contrast, all these citrus strains were found to extensively colonize the apoplast of Mexican lime leaves [26]. These events suggest the requirement for chemotaxis and active bacterial motility on the plant surface to locate and colonize the apoplastic site.

Studies have elucidated some of the pathogenesis mechanisms that contribute to host range differences in CBC strains [27,28,29,30], but they did not address, most of the time, early events in the infection process, including motility mediated by chemotaxis [31].

In this study, we characterized the chemotactic responses of types A and A* or A^w^ of *Xcc* and compared their behavior with *Xec*, the causal agent of citrus bacterial spot (CBS), a disease of citrus nursery plants, and *Xc*, the cause of crucifer black rot (CBR) and whose chemotactic role in leaf colonization has been demonstrated [16]. Our aim was to identify the profiles of compounds that act as attractants or repellents for *Xanthomonas* strains and to relate these profiles to carbon source use, MCP content and host range. Furthermore, the chemotactic response to apoplastic fluids from citrus and non-citrus hosts was evaluated in order to determine whether the chemotaxis signals may somehow explain the host specificity of *Xanthomonas* strains at an early stage of infection.

## 2. Materials and Methods

### 2.1. Bacterial Strains, Culture Media, and Growth Conditions

Representative bacterial strains from each xanthomonad group used in this study and their natural hosts are listed in Table 1. Two wide-host-range strains (A type) of *Xanthomonas citri* pv. *citri* (*Xcc*) and three narrow-host-range strains (A* and A^w^) were evaluated along with *X. euvesicatoria* pv. *citrumelonis* (*Xec*) and *X. campestris* pv. *campestris* (*Xc*), a non-citrus pathogen.

Bacterial strains were routinely grown on Luria Bertani broth (LB; 10 g of tryptone, 5 g L^−1^ of yeast extract, and 5 g of sodium chloride) or on LB plates (1.5% bacteriological agar) at 27 °C for 48 h.

### 2.2. Carbon Source Use by Xanthomonas Strains

Biolog GN2 MicroPlateTM was used for analysis of carbon source use following the manufacturer’s instructions (Biolog Inc. Hayward, CA, USA). Bacterial strains were grown on LB agar plates and incubated for 48 h at 27 °C. Bacterial colonies were then harvested and suspended in 0.85% NaCl and adjusted to 0.3 absorbance at 600 nm. Each Biolog microplate well was seeded with 150 µL of the bacterial suspension and incubated for 24 h at 27 °C without shaking. Tetrazolium oxidation activity was measured at 0 and 24 h in a microplate reader set at 570 nm absorption.

The assay was repeated at least two times with two replicates per assay. Carbon source use was calculated by subtracting the time 0 absorbance from each well reading. Substrate well readings were further adjusted against the substrate blank well, and each activity value was the average of the assays, with two replicates per assay. Wells with ≥160% of activity compared to the blank were considered positive and ≤130% of activity considered negative. Values from 129% to 159% were considered non-informative and dropped from further analysis. Data from informative and discriminatory tests were converted to binary form, and similarity coefficients for pairs of strains were calculated with PAST v.4.03 software (University of Oslo, Oslo, Sweden) [32] using the Jaccard coefficient and subjected to the unweighted pair group method (UPGMA). Bootstrap values (based on 1000 replicates) were indicated at the nodes.

### 2.3. Chemotactic Response of Xanthomonas Strains to Carbon Compounds

A new microtiter plate assay was developed based on a capillary protocol previously described [33]. Pipette tips containing 5 µL of the carbon source were inserted into 48 wells of a microtiter plate, each filled with 200 µL of a 10^8^ CFU mL^−1^ bacterial suspension. To measure chemotaxis, the number of bacteria able to enter the tip for 1 hour was estimated by means of serial dilutions of the tip’s content. Bacteria used in chemotaxis studies were in the logarithmic phase to ensure active motility; briefly, a colony was harvested from the LB plate, suspended in 5 mL of LB broth, and incubated at 27 °C and shaking o/n at 150 rpm, and then this preculture was diluted in 30 mL of LB broth to a final concentration of 0.01 OD at 600 nm and cultured up to the logarithmic phase in the conditions described before. Bacteria were washed twice with 10 mM MgCl_2_. The carbon source was considered a chemoattractant or chemorepellent when the average number of bacteria that entered the tip in six replicates from at least two assays was significantly higher or lower (*p* < 0.05) than the control with 10 mM MgCl_2_. The assay was validated using *D. dadantii* strain 3937, the causal agent of potato soft rot, whose chemotactic profile has been previously described [14,34]. Data from the microtiter plate assay were converted to binary form, and similarity coefficients for pairs of strains were calculated, as described before.

### 2.4. Chemotactic Response of Xanthomonas Strains to Apoplastic Fluids

Apoplastic fluids were extracted, as previously described for *Solanum lycopersicum* [35]. Briefly, weighed leaves were vacuum-infiltrated with sterile distilled water, introduced into a 5 mL tip, and then centrifuged at 4000× *g* for 20 min. After centrifugation, the suspension containing the apoplastic fluid was recovered in 1.5 mL tubes and centrifuged at 3000× *g* to remove leaf debris. Apoplastic fractions were sterilized by passing them through a 0.2 µm filter. To evaluate the effect of leaf apoplastic fluids, microtiter plate assays were performed, as described before, with fractions of 200, 100, 50, 12.5, 6.3, and 3.1 mg of leaf per mL of sterile distilled water.

The experiment was performed using the microtiter plate assay described in the previous section. To establish an apoplastic fluid threshold concentration for every strain separately, data were analyzed using the Dunnet test on JMP software (SAS Institute Inc., Cary, NC, USA); this test compares using a *t*-test every apoplastic concentration with the control, the homogeneous environment (water in this experiment.) The apoplastic fluid concentration was considered a chemoattractant or chemorepellent when the average number of bacteria that entered the tip in six replicates from at least two assays was significantly (*p* < 0.05) higher or lower than the water control. When *p* > 0.05, no response was considered.

### 2.5. Detection of Methyl-Accepting Chemotaxis Proteins

Profiles of MCPs for *Xanthomona*s species and pathovars used in this study were determined *in silico* based on the analysis of a selection of complete representative genomes from the database of each xanthomonad group studied (Table 2) and the search of homologous sequences for 28 MCPs available, as previously described [19]. Sequence homology searches were conducted using Geneious Prime v.2022.1.1 (Biomatters, Auckland, New Zealand).

To classify xanthomonads studied according to their MCP profile, cluster analysis was performed as before, using PAST v.4.03 software (University of Oslo, Oslo, Sweden) [32].

To confirm the MCP content in xanthomonads used in this study, conventional PCR was conducted according to the genomic analysis and using selected primers previously described for MCPs that were not conserved and showed variability within the *Xanthomonas* genus such as XAC3271, XAC3768, XCV1702, XCV1778, XCV1942, XCV1944, XCV1947, XCV1951, and XCC0324 [19]. Two extra set of primers were designed based on the genes XCAW2504 (MSV_XCAW2504F: ATGCTGTCGGAAATGCAGGA and MSV_XCAW2504R: AGGTGCTTGATCTCCTTGGC) and XCAW2508 (MSV_XCAW02508F: GCGTCGCTCAATAACGTCAC and MSV_XCAW02508R: GATGCTGCTTTCGTACTGCG) that were identified in *Xcc* 12879 and corresponded to XCV1933 and XCV1938, which primers described previously [19] did not give positive results from some *Xcc* A strains in a preliminary work in our group. PCR was carried out in a final volume of 25 µL containing 2 mM MgCl_2_, 0.2 mM of dNTPs (each), 2 units of DNA polymerase (Biotools, Madrid, Spain), and 0.2 mM of each primer. For fragments longer than 1000 bp, FastStart Taq-DNA polymerase from (Roche, Basel, Switzerland) was used to a final volume of 25 µL containing 2 mM of MgCl_2_, 0.1 mM of dNTPs (each), 2 units of FastStart Taq-DNA polymerase, and 0.2 mM of each primer. The amplification conditions consisted of 94 °C for 1 min, annealing temperatures described by Mhedbi-Hajri [19] and 57 °C for XCAW2504 and XCAW2508 for 1 min and 72 °C for 1 min for 40 cycles, plus an initial step of 95 °C for 10 min and a final step of 72 °C for 10 min. PCR products (10 µL) were run in 1.5% (*w*/*v*) agarose gels stained with ethidium bromide and visualized under a UV transilluminator. Water was used as a negative control. The presence or absence of the PCR product for each MCP was converted to binary form and cluster analysis performed, as described before.

## 3. Results

### 3.1. Carbon Source Use by Xanthomonas Strains

Carbon source use was analyzed for bacterial strains listed in Table 1 with Biolog GN2 Microplate TM (Biolog Inc. Hayward, CA, USA) following the manufacturer instructions. Readings were made at 0 and 24 h post-inoculation (hpi) to detect the earliest metabolic response. The use of carbon sources that differentiate *Xanthomonas* strains studied is presented in Table 3.

Tween 40, N-acetyl-D-glucosamine, D-cellobiose, D-fructose, D-galactose, gentiobiose, α-D-glucose, maltose, D-mannose, D-psicose, D-trehalose, pyruvic acid methyl-ester, α-keto glutaric acid, succinic acid, bromosuccinic acid, L-glutamic acid, L-proline, L-serine, L-threonine, and glycerol were used by all strains tested. The compounds not metabolized by any of the strains were α-cyclodextrin, N-acetyl-D-galactosamine, adonitol, m-inositol, D-mannitol, β-methyl-D-glucoside, L-rhamnose, D-sorbitol, xylitol, citric acid, formic acid, D-galactonic acid lactone, D-galacturonic acid, D-glucosaminic acid, D-glucuronic acid, γ-hidroxybutyric acid, ρ-hydroxy phenylacetic acid, itaconic acid, quinic acid, sebacic acid, glucuronamide, L-histidine, L-leucine, L-ornithine, L-phenylalanine, L-pyroglutamic acid, D-serine, D,L-carnitine, γ-amino butyr acid, inosine, thymidine, phenyethyl-amine, and 2-aminoethanol. Compared with *Xc*, citrus strains used cis-aconitic acid and L-alanyl-glycine. *Xc* specifically used Tween 80, D-saccharic acid, and uridine. Among the citrus strains, *Xcc* A, A*, and A^w^ strains used sucrose. *Xcc* 306 was atypical compared with all other *Xcc* A strains in that no activity was detected for dextrin, L-fucose, lactulose, and α-keto butyric acid; meanwhile, *Xcc* A* Iran10 was the only strain that responded to uridine. Glycyl-L-aspartic acid, propionic acid, D-alanine, and L-alanine were used by A* and A^w^ but not by wide-host-range A strains. *Xcc* A* strains were the only one that responded to α-D-lactose, turanose, and L-aspartic acid. In addition, *Xcc* A*, as did *Xec* and *Xc*, used D-melobiose, α-hydroxybutiric acid, and D,L-lactic acid.

**Figure 1:**
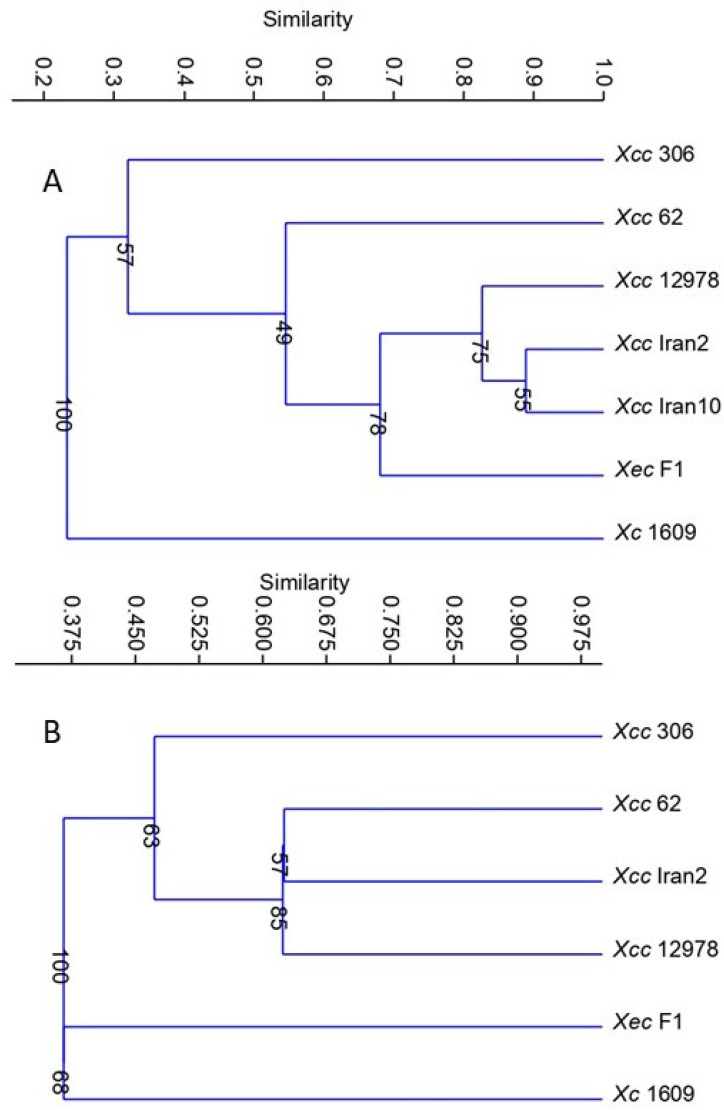
Dendrograms showing relationships among *Xanthomonas* strains based on data from (**A**) Biolog GN2 activity and (**B**) chemotaxis assay.

To study the overall relatedness of the metabolic response among the xanthomonads evaluated, cluster analysis was performed by transforming the data from carbon source use to binary form (uninformative carbon sources were dropped from the analysis). The analysis demonstrated that citrus strains were grouped in the same cluster and separated from *Xc*. Moreover, *Xcc* A strains were clustered according to the host range, i.e., separated from strains *Xcc*, A*, A^w^, and *Xec* (Figure 1A), and the two *Xcc* strains showed their diversity.

Because a possible relationship between carbon source use and host range was elucidated, the putative role of chemical compounds in chemotaxis was studied later.

### 3.2. Chemotactic Response of Xanthomonas Strains to Carbon Compounds

To define the chemotactic profile of *Xanthomonas* strains, a new chemotaxis assay, in which several compounds were concurrently tested with a large number of technical replicates, was developed. In this assay, the quantity of bacteria entering a pipette tip containing the carbon source was used to assess the chemotactic response independently of bacterial growth. This experimental approach has the same principle as the protocols described previously [33,36,37]. This assay was validated with *D. dadantii* 3937, and the chemotactic response obtained matched those previously reported: 10 mM cysteine was repellent, and 10 mM sodium citrate, 10 mM glucose, and 1 and 200 mM serine were attractants [14,34].

To determine the chemotactic responses of the *Xanthomonas* studied, 19 compounds were tested (see Table 4); from these chemicals, the metabolic response was determined using Biolog GN2 for 14 of them, and therefore, solely sodium citrate, xylose, arginine, cumaric acid, and cysteine’s metabolic responses were not considered.

All *Xanthomonas* strains evaluated responded similarly to 10 mM cysteine as a repellent and 10 mM sucrose, 0.2% glycerol, and 200 mM serine as attractants (Table 4).

Interestingly, the repellent cysteine has not been detected in the phloem sap of most *Citrus* spp. [38], and sucrose was previously reported as an attractant for other *Xanthomonas* spp. [36,39]. *Xc* differed from citrus strains in that 10 mM alanine and 10 mM leucine acted as repellents and 10 mM glucuronic acid as an attractant. The responses that differentiated *Xcc* strains from *Xec* and *Xc* were 150 mM leucine and 0.2% mannitol as attractants for *Xcc* strains (repellent for *Xec* and no response for *Xc*) and 10 mM xylose and 10 mM serine as repellents for *Xec* and *Xc*, while no response was observed for *Xcc* strains. In addition, among citrus pathogenic strains, *Xec* was the sole strain showing a repellent response toward fructose and glucose, two well-known carbon sources for bacteria; 200 mM alanine did not show any chemotactic effect in *Xec*, while it was an attractant for the *Xcc* strains tested. As previously reported for *Ralstonia solanacearum* strains [40], chemotactic responses varied within *Xcc* strains; *Xcc* 306 and *Xcc* 62 were the only strains attracted to 10 mM galacturonic acid, along with *Xec*. *Xcc* 62 was the only *Xcc* A strain attracted to 10 mM arginine, and *Xcc* 306 was the only showing no response to 100 mM arginine or being attracted by leucine at 10 mM. Cluster analysis based on chemoattraction grouped *Xcc* A strains with the narrow-host-range strains *Xcc* A* Iran2 and *Xcc* A^w^ 12879 and separated them from *Xec* and *Xc.* Within the *Xcc* subgroup, *Xcc* 62 was more closely related to *Xcc* A* Iran2 and *Xcc* A^w^ 12879 than to *Xcc* 306 (Figure 1B). Chemotactic responses were more similar for narrow-host-range strains, while the wide-host-range strains responses were variable.

### 3.3. Identification of MCPs in Xanthomonas Species Used in the Study

**Figure 2:**
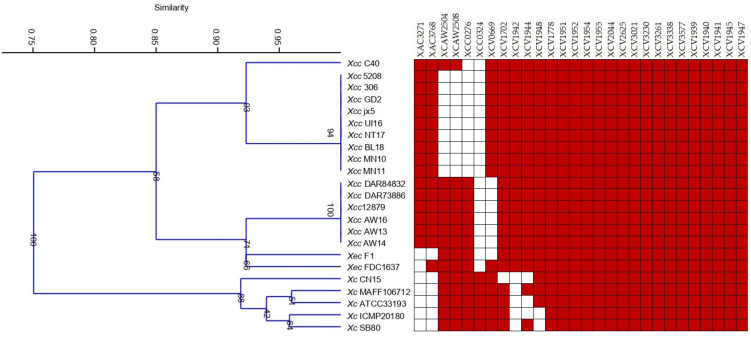
Dendrogram and heat map resulting from MCP identification in 23 completed genome sequences from *Xcc*, *Xce*, and *Xc* strains described in Table 2. Red colour in the heat map means presence of the MCP in the strain.

The analysis of the complete genomes of different *Xanthomonas* species, pathovars, and pathotypes revealed variants in their MCP profiles. Although 28 different MCPs were found in the genome sequences, the number of MCPs varied from 24 in most of the type A *Xcc* strains to 26 in all A*/A^w^ *Xcc* and *Xec* FDC1637 strains. An MCP pattern composed of 18 genes was shared by all genomes analyzed; meanwhile, citrus-associated and brassica-associated strains shared 22 and 24 MCPs, respectively. Among those common MCPs, XCV1942, XAC3768, and XAC3271 were only present in citrus-associated xanthomonads and XCC0324 was only found in brassica-associated ones (Figure 2). Results also showed that the MCP content differed among citrus xanthomonads; thereby, XAC3271 was only identified in *Xcc*, but it was not found in *Xec*, and although XCAW2504 and XCAW2508 were detected in all *Xcc* A*/A^w^ strains, they were found in just one *Xcc* A strain and were not identified in any *Xec*.

Cluster analysis of the binary data obtained from MCP analysis revealed major groups according to pathotype and *Xanthomonas* spp. (Figure 2). One cluster included all *Xcc* type A strains separated from A^w^/A* that grouped together with *Xec* and more separated from *Xc* (Figure 2).

PCR using primers previously described [19] in addition to those for XCAW2504 and XCAW2508 results confirmed findings from the genomic analysis (Table 5). XCV1942, XAC3768, and XAC3271 were identified in citrus strains but not in *Xc* 1609, and XCC0324 was only found in *Xc* 1609. In addition, some other MCPs were universally distributed in all the strains, in line with genomic results. As well, either A* or A^w^ *Xcc* strains showed the same MCP/PCR profile; meanwhile, variability among *Xcc* A strains was found in the MCP content (Figure 2, Table 5).

The difference in the presence of specific MCPs was related to the host (citrus vs. crucifer) and the citrus pathogenic species (*Xec* vs. *Xcc* strains); moreover, the minor differences revealed within the *Xcc* A strains were in concordance with their different chemotactic responses to carbon compounds.

### 3.4. Xanthomonas Strains Are Attracted by Leaf Apoplastic Fluids

To confirm the role of chemotaxis at an early stage of leaf infection, chemotaxis of the different strains was assessed in response to apoplastic fluids from sweet orange (*C. sinensis*) var. ‘Valencia Late’, Mexican lime (*Citrus aurantifolia*), and Chinese cabbage (*Brassica pekinensis*) var. Kasumi. Our results showed that all apoplastic fluids act as chemoattractants (Table 6). Both cabbage and citrus apoplastic fluids were attractive for all *Xanthomonas* strains.

**Figure 3:**
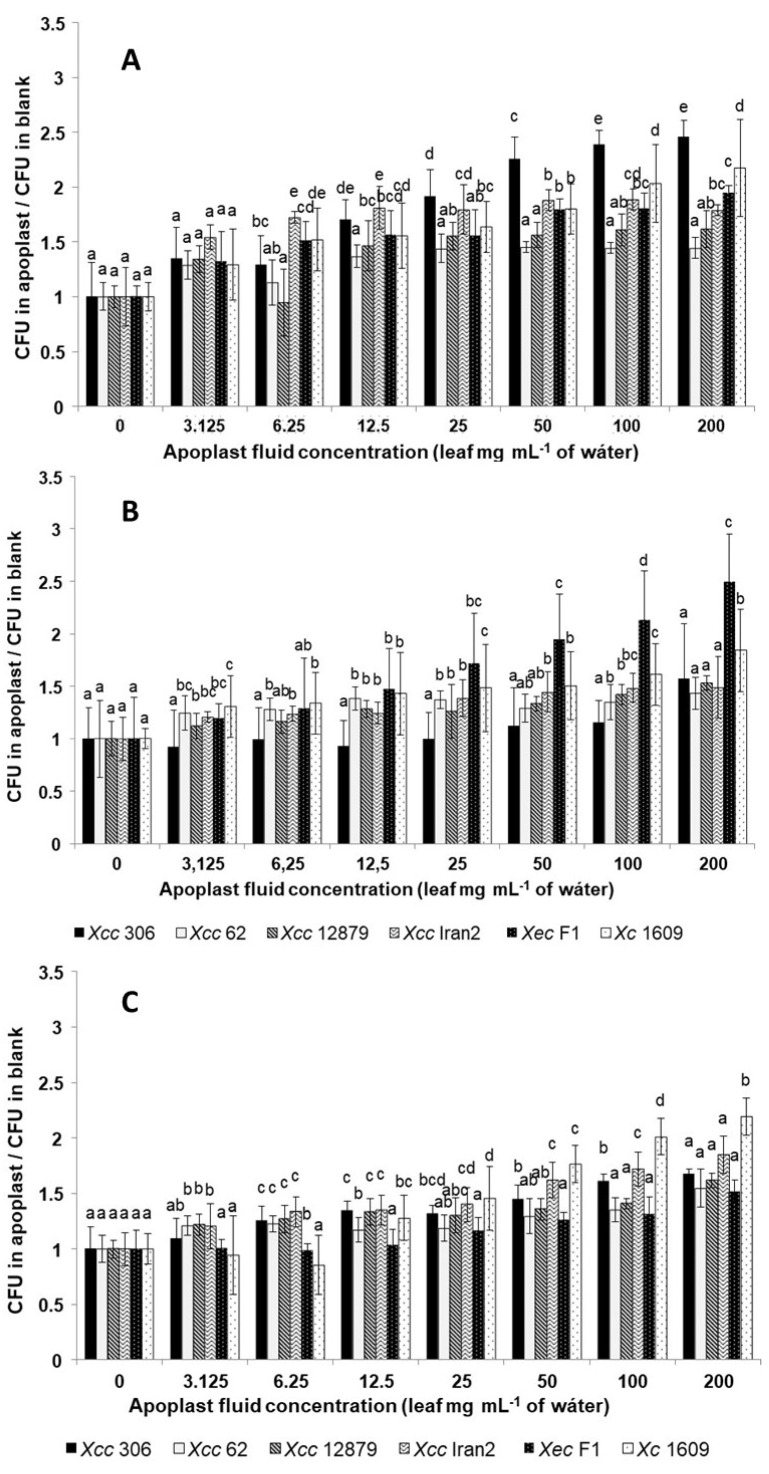
Comparison of the chemotactic response of different *Xanthomonas* strains to apoplastic fluid extracts from leaves of (**A**) sweet orange, (**B**) Mexican lime and (**C**) Chinese cabbage. The graphs show the relative number of bacteria entering the tip in the presence of an apoplastic fluid at different concentrations. The graph shows the mean with the standard deviation (error bars). Means with the same letter within a sample do not differ significantly (*p* < 0.05).

Nevertheless, the response differed among strains: *Xcc* A 306 was more responsive to sweet orange, *Xec* F1 to Mexican lime, and *Xc* 1609 to Chinese cabbage, indicating a clear difference in the response between citrus and crucifer strains (Figure 3). Moreover, although these strains weakly responded to the lowest concentrations of apoplast fluids from these species (Table 6), their chemoattractive response increased markedly with the apoplastic fluid concentration. The same occurred for the interaction between *Xcc* A 306 and Mexican lime (Figure 3).

**Figure 4:**
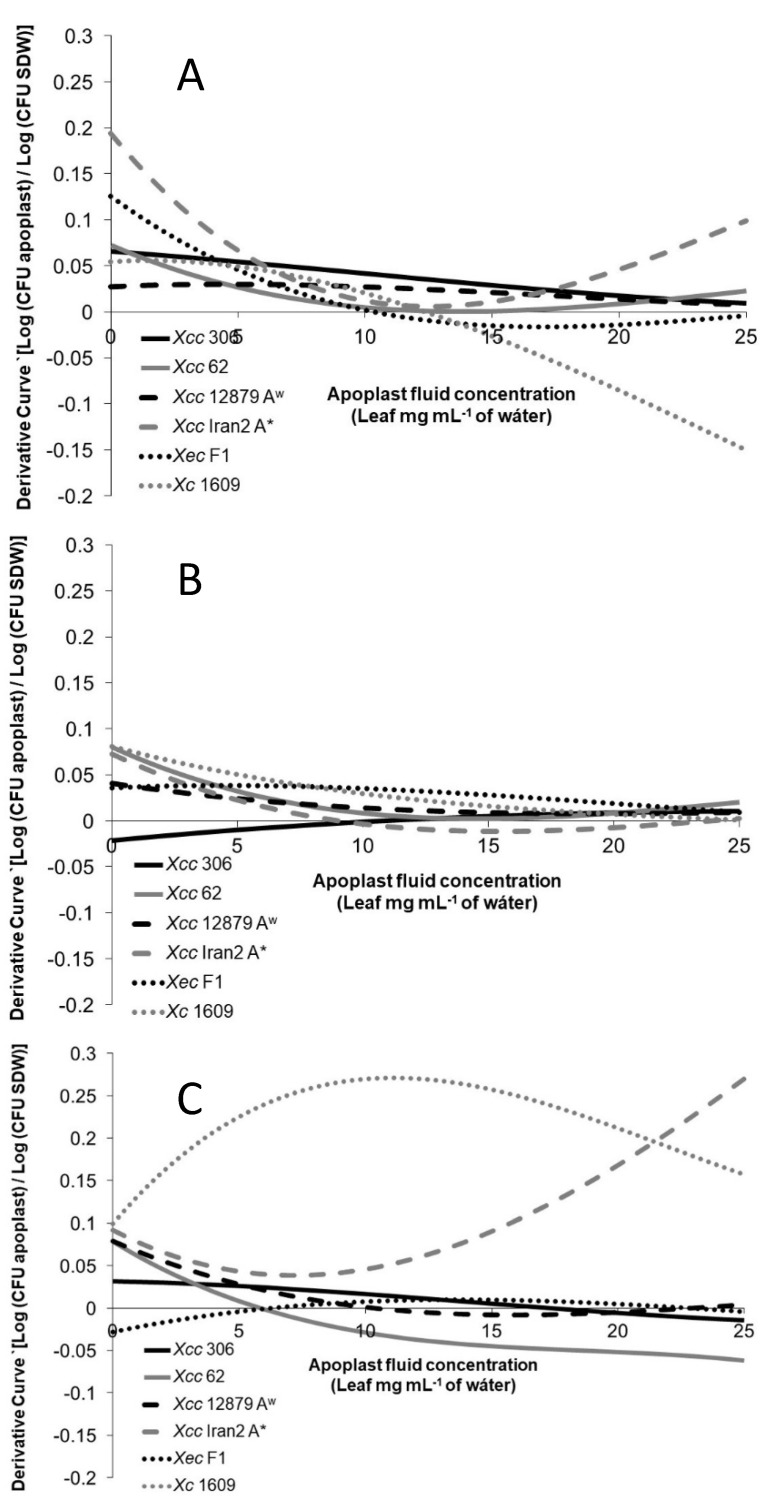
Variation of the chemotaxis response of *Xanthomonas* strains toward leaf apoplastic fluids at different concentrations (from 0 to 25 leaf mg mL^−1^) from (**A**) sweet orange, (**B**) Mexican lime, and (**C**) Chinese cabbage. The graphs show the derivative curve of the regression curve obtained from data of the chemotaxis assay explained before. The chemotactic responses of the xanthomonads strains fitted onto polynomial regression curves (r^2^ > 0.8) were derived and the curves plotted. Herein, apoplastic fluid concentrations from 0 to 25 mg mL^−1^ were selected because almost no variation (*p* > 0.05) was observed at higher concentrations, due to a possible saturation of chemoreceptors.

To evaluate more precisely the differences among the strains on the different hosts, the variation of the chemotactic response related to the apoplast concentration increase was analyzed. The chemotactic derivative curves in Figure 4 show how the chemotactic response changed as the apoplastic fluid concentration increased. 

The chemotactic responses of the *Xanthomonas* strains tested toward citrus apoplastic fluids (Figure 4A,B) showed higher response changes at low concentrations for most of the strains. Usually, the chemoattractive response diminished or even declined as the apoplastic fluid concentration increased. However, it is important to note that this reduction in the chemoattractive response does not mean a negative response (chemorepellent) but fewer bacterial cells entering the tip with apoplastic concentration increments.

Citrus pathogenic strains’ response toward the Chinese cabbage apoplastic fluid was constant or even negative when the concentration increased, with the exception of *Xcc* Iran2 A* (Figure 4C). The same behavior was observed in *Xc* 1609 toward most citrus apoplastic fluids. This result suggests that on a non-host-plant leaf surface, the xanthomonad chemotactic response would not be as efficient as the pathogen approaches the stomata.

The orange leaf apoplastic fluid produced the most variable response among the *Xanthomonas* strains tested (Figure 4A). The highest variation of the response associated with the concentration was observed for *Xcc* 306, presenting *Xec* F1 and *Xcc* Iran2, A* an intermediate phenotype; meanwhile, the lowest variation was found in *Xcc* 62 and *Xcc* A^w^ 12879. Moreover, the response of *Xcc* Iran2 A* showed a reduction in the variation at concentrations over 6.25 mg mL^−1^.

No differences in the chemotactic response toward Mexican lime was observed among *Xcc* 62, *Xcc* A^w^ 12879, and *Xcc* Iran2 A* (Figure 4B). However, less reaction was observed for *Xcc* 306, although the response increased with the apoplastic fluid concentration.

*Xc* 1609 was highly responsive toward the Chinese cabbage apoplastic fluid compared with citrus *Xanthomonas* (Figure 4C) and less reactive to citrus apoplastic fluids (Figure 4A,B).

## 4. Discussion

Chemotaxis plays a key and early role in bacterial attachment, biofilm development, and bacterial regulation in response to the environment [21,41,42,43,44]. Moreover, in previous studies, chemotaxis in *Xcc* has been described as a central plant colonization factor at the early stages of the microbe–plant interaction [44,45]. In addition, biofilm formation has been reported as an important step for citrus canker establishment and for *Xcc* to survive on the plant surface [25,46]. In addition, the ability of xanthomonads to form biofilm on citrus has been associated with the host range [26].

To look for the possible link between the chemotactic response and the xanthomonad host range, the metabolic activity on carbon sources was compared to the chemotactic response as well as to the MCP content on *Xcc* pathotypes, *Xec* and *Xc*. The study first showed that, interestingly, CBC wide-host-range strains were able to metabolize fewer metabolites than the narrow-host-range strains. This low ability to metabolize carbon compounds may involve a restriction regarding the environment in which bacteria can multiply and, for instance, a stronger need to colonize the apoplastic space to meet nutritional requirements not available on the leaf surface. This niche restriction might make wide-host-range strains evolve different strategies, such as chemotaxis and virulence factors, to colonize the citrus host interior in order to get access to their nutritional requests. On the contrary, narrow-host-range strains, with higher metabolic capacity, would not require all the same abilities. In previous works by our group, differences in biofilm formation and swimming motility were shown between narrow- and wide-host-range strains of *Xcc*, and this may be related to their different nutritional requests [26].

Our results showed variable chemotaxis responses among the *Xanthomonas* strains tested according to their host range and similar clustering from either their overall metabolic activity or chemotaxis toward chemical compounds, noting that chemotaxis responses in xanthomonads described here might be metabolism dependent in response to effectors addressed to alter energy metabolism or increase intracellular energy. Further analysis is needed to determine the specific role in chemotaxis of particular compounds, their putative synergistic effects, and the impact of their relative concentrations.

*Xcc*, as many other *Xanthomonas* strains, goes through an epiphytic phase from leaf-deposition until reaching the apoplast [20,31]. During this stage, bacterial sensors, such as MCPs, among others, teach and guide the bacteria where they are and where to go. Herein, the MCP content of *Xcc*, *Xec*, and *Xc* was determined based on the data of available genomes and, besides, partially confirmed by PCR in strains used in the study. Cluster analysis based on the MCP content of *Xcc*, *Xec*, and *Xc* profiles from the complete genome resulted in groups according to the strain host and therefore also according to carbon source use and chemotactic profile toward chemicals. Moreover, differences in the MCP profile among closely related strains, such as *Xcc* pathotypes, were elucidated, resulting in different groups according to the host range in cluster analysis. All the dendrograms from metabolic activity, chemotactic response, or MCP content showed a clear difference between the citrus pathogenic strains and the crucifer black rot strain, as well as changes between wide- and narrow-host-range *Xcc* strains.

The *Xanthomonas* response toward apoplastic fluids of strains with different MCP profiles showed a unique response toward sweet orange, Mexican lime, and Chinese cabbage leaf apoplastic fluids according to the host range of the xanthomonads evaluated. Strains *Xcc* 62, *Xcc* 12879, and *Xcc* Iran2, which responded similarly to apoplastic fluids, showed more similar MCP profiles based on PCR results. It should be noted that strain *Xcc* 62, closely related to *Xcc* 306, presented a chemotactic profile on PCR closer to narrow-host-range CBC strains than to *Xcc 3*06. However, strains *Xcc* 62, *Xcc* 12879, and *Xcc* Iran2, even showing the same MCP content on the PCR profile, presented a variable chemotaxis response. This apparent incongruence may be because the limitation of MCP analysis with PCR that was not able to entirely determine the MCP content in these strains due to variability in the PCR primer target sequence or because their chemotaxis may be mediated by several mechanisms besides MCPs, which are variable among CBC strains with different host ranges [47,48,49]. However, differences in the MCP content among A pathotype strains was supported by the results of genomic analysis performed here, which showed “atypical” profiles in some strains within *Xcc* type A.

Our results suggest that apoplastic fluids, exuding from stomatal openings or leaf wounds, are likely to act as a whole or contain specific chemotactic signals, currently not identified, that would determine the behavior of the pathogen on the leaf surface. These results, along with those from other authors [16,19,21,44], support the role of chemotaxis in the plant–bacteria interaction and the *Xanthomonas* host range. Moreover, our findings are consistent with those in other models, such as *R. solanacearum*, which is more attracted to host root exudates than to non-host exudates [40], or *X. oryzae*, which is attracted toward root exudates based on the susceptibility of the rice cultivar [36]. Our study confirms that apoplastic fluids exuding from stomatal openings or leaf wounds would be detected by the bacteria and that they will trigger a host-dependent chemotactic response leading these xanthomonads toward the host entrances. The apoplastic fluid from the substomatal cavity might be diluted by the natural humidity on the leaf, especially after a raining event that transports the bacteria from one tree to the other, facilitating the bacteria–apoplastic fluid interaction. On the leaf surface, the bacteria would move toward increasing concentrations of the apoplastic fluid as they approach the stomata or wounds. However, at high apoplastic fluid concentrations, as occurring within the apoplast, motility is no longer needed and biofilm formation and effector secretion into plant cells are prompted [26,44,46,50].

To conclude, our work supports the links between the host range of citrus pathogenic *Xanthomonas* strains, the use of carbon sources, and the chemotaxis response to these carbon sources or host leaf apoplastic fluids. Our results indicate a role of leaf apoplastic exudates in chemotaxis and their involvement in the early stages of bacterial infection and host range processes. However, further investigation is needed to determine specific components of the apoplast that underline the chemotaxis mechanism in citrus species or the role of different environmental sensors, including MCPs, in it.

## Figures and Tables

**Table 1:** Strains and hosts of *Xanthomonas* spp. used in the study.

Strain	Taxon, Disease and Disease Type	Natural Host
*Xcc* 306	*Xanthomonas citri* pv. *citri*, CBC ^a^ A	*Citrus sinensis*
*Xcc* 62	*Xanthomonas citri* pv. *citri*, CBC A	*Citrus paradisi*
*Xcc* Iran2	*Xanthomonas citri* pv. *citri*, CBC A*	*Citrus aurantifolia*
*Xcc* Iran10	*Xanthomonas citri* pv. *citri*, CBC A*	*Citrus aurantifolia*
*Xcc* 12879	*Xanthomonas citri* pv. *citri*, CBC A^w^	*Citrus aurantifolia*
*Xec* F1	*Xanthomonas euvesicatoria* pv. *citrumelonis,* CBS ^b^	*Citrus* spp.
*Xc* 1609	*Xanthomonas campestris* pv. *campestris*, CBR ^c^	*Brassica* spp.

^a^ CBC: citrus bacterial canker; ^b^ CBS: citrus bacterial spot; ^c^ CBR: crucifer black rot.

**Table 2:** Genomes used for in silico MCP analysis.

Strain	Species/Pathovar	Type	Accession/Assembly
*Xcc* C40	*X. citri* pv. *citri*	A	CCWX01
*Xcc* 5208	*X. citri* pv. *citri*	A	NZ_CP009028.1
*Xcc* 306	*X. citri* pv. *citri*	A	NC_003919.1
*Xcc* gd2	*X. citri* pv. *citri*	A	NZ_CP009019.1
*Xcc* jx5	*X. citri* pv. *citri*	A	NZ_CP009010.1
*Xcc* UI6	*X. citri* pv. *citri*	A	NZ_CP008990.1
*Xcc* NT17	*X. citri* pv. *citri*	A	NZ_CP008993.1
*Xcc* BL18	*X. citri* pv. *citri*	A	NZ_CP009023.1
*Xcc* MN10	*X. citri* pv. *citri*	A	NZ_CP009002.1
*Xcc* MN11	*X. citri* pv. *citri*	A	NZ_CP008999.1
*Xcc* DAR73886	*X. citri* pv. *citri*	A*	GCA_016801635.1
*Xcc* DAR84832	*X. citri* pv. *citri*	A*	GCA_016801615.1
*Xcc* 12879	*X. citri* pv. *citri*	A^w^	NC_020815.1
*Xcc* AW13	*X. citri* pv. *citri*	A^w^	NZ_CP009031.1
*Xcc* AW14	*X. citri* pv. *citri*	A^w^	NZ_CP009034.1
*Xcc* AW16	*X. citri* pv. *citri*	A^w^	NZ_CP009040.1
*Xec* F1	*X. euvesicatoria* pv. *citrumelonis*	NA^b^	GCA_000225915.1
*Xec* FDC1637 ^a^	*X. euvesicatoria* pv. *citrumelonis*	NA	GCA_005059795.1
*Xc* CN15	*X. campestris* pv. *campestris*	NA	GCA_000403575.2
*Xc* MAFF302021	*X. campestris* pv. *campestris*	NA	GCA_009177345.1
*Xc* ATCC33193	*X. campestris* pv. *campestris*	NA	GCA_000007145.1
*Xc* ICMP20180	*X. campestris* pv. *campestris*	NA	GCA_001186415.1
*Xc* SB80	*X. campestris* pv. *campestris*	NA	GCA_021459985.1

^a^ All sequences corresponded to full complete genomes, except *Xec* FDC1637, which enclosed 124 contigs. ^b^ Not applicable (NA).

**Table 3:** Biolog activity of carbon sources that differentiates species and strains of *Xanthomonas* pathogenic for citrus and crucifers.

Strains/Additive ^a^	*Xcc* 306	*Xcc* 62	*Xcc* 12879	*Xcc* Iran2	*Xcc* Iran10	*Xec* F1	*Xc* 1609
Dextrin	−	+	+	+	+	+	+
Glycogen	+ ^a^	+	NI	+	+	+	NI
Tween 80	− ^b^	−	−	−	−	−	+
L-Arabinose	−	−	−	−	NI	NI	−
D-Arabitol	−	−	−	−	−	NI	−
L-Fucose	NI ^c^	+	+	+	+	+	+
α-D-Lactose	−	−	−	+	+	NI	−
Lactulose	NI	+	+	+	+	+	+
D-Melobiose	−	NI	−	+	+	+	+
D-Raffinose	−	−	−	NI	NI	NI	NI
Sucrose	+	+	+	+	+	NI	+
Turanose	−	−	−	+	+	NI	NI
Succinic Acid Mono-Methyl-Ester	NI	+	+	+	+	+	+
Cis-Aconitic Acid	+	+	+	+	+	+	−
D-Gluconic Acid	−	−	−	−	NI	−	−
α-Hidroxybutyric Acid	−	−	NI	+	+	+	+
β-Hidroxybutyric Acid	−	−	−	−	NI	NI	NI
α-Keto Butyric Acid	−	+	+	+	+	+	+
D,L-Lactic Acid	−	−	NI	+	+	+	+
Malonic Acid	NI	NI	+	+	+	+	+
Propionic Acid	−	−	+	+	+	−	+
D-Saccharic Acid	−	−	−	−	−	−	+
Succinamic Acid	NI	+	+	+	+	+	NI
L-Alaninamide	+	+	+	+	+	+	NI
D-Alanine	−	−	+	+	+	+	−
L-Alanine	NI	−	+	+	+	+	NI
L-Alanyl-Glicine	+	+	+	+	+	+	−
L-Asparagine	−	−	NI	−	NI	−	−
L-Aspartic Acid	−	−	NI	+	+	−	NI
Glycyl-L-Aspartic Acid	−	−	+	+	+	−	−
Glycyl-L-Glutamic Acid	+	+	+	+	+	+	NI
Hydroxy-L-Proline	−	−	−	−	NI	−	NI
Urocanic Acid	−	−	−	−	NI	−	−
Uridine	−	−	−	−	+	−	+
D,L-α-Glycerol Phosphate	−	−	NI	+	+	+	−
α-D-Glucose-1-Phosphate	−	NI	−	+	+	NI	−
D-Glucose-6-Phosphate	−	−	−	+	+	+	−

Wells with ≥160% of activity at 24 h compared to the blank were considered positive (+) ^a^ and ≤130% of activity considered negative (−) ^b^. Values from 129% to 159% were considered non-informative and dropped from further analysis (NI) ^c^.

**Table 4:** Chemotactic response of the species and strains of *Xanthomonas* pathogenic on citrus and crucifers.

Additive	*Xcc* 306	*Xcc* 62	*Xcc* Iran2 A*	*Xcc* 12879 A^w^	*Xec* F1	*Xc* 1609
Sodium Citrate 10 mM	+ ^a^	0 ^b^	+	+	0	0
Fructose 10 mM	0	+	0	+	− ^b^	0
Galactose 10 mM	0	+	0	+	0	+
Glucose 10 mM	0	0	0	+	−	0
Maltose 10 mM	0	+	+	+	0	+
Sucrose 10 mM	+	+	+	+	+	+
Xylose 10 mM	0	0	0	0	−	−
Arginine 10 mM	0	+	0	0	0	0
Arginine 100 mM	0	+	+	+	+	+
Alanine 10 mM	0	+	+	0	0	−
Alanine 250 mM	+	+	+	+	0	+
Cysteine 10 mM	− ^c^	−	−	−	−	−
Leucine 10 mM	+	0	0	0	0	−
Leucine 150 mM	+	+	+	+	−	0
Serine 10 mM	0	0	0	0	−	−
Serine 200 mM	+	+	+	+	+	+
Glycerol 0.2%	+	+	+	+	+	+
Mannitol 0.2%	+	+	+	+	−	0
Galacturonic Acid 10 mM	+	+	0	0	+	0
Glucuronic Acid 10 mM	0	0	0	0	0	+
Citric Acid 10 mM	−	0	0	−	0	0
Succinic Acid 10 mM	0	+	+	0	+	0
Cumaric Acid 10 mM	0	+	0	+	+	0

^a^ Chemoattractant (+); ^b^ no response; ^c^ chemorepellent (−).

**Table 5:** PCR amplification of some xanthomonads’ MCPs using primers previously described [19] and those designed in this study.

Strains/Primers		*XAC*3271	*XAC*3768	XCCAW2504	XCCAW2508	*XC*V1702	*XC*V1778	*XC*V1942	*XC*V1944	*XC*V1947	*XC*V1951	XCC0324
CBC ^a^ A type	*Xcc* 306											
	*Xcc* 62											
CBC A^w^ type	*Xcc* 12879											
CBC A* type	*Xcc* Iran2											
	*Xcc* Iran10											
CBS ^b^	*Xec* F1											
CBR ^c^	*Xc* 1609											

^a^ CBC: citrus bacterial canker, ^b^ CBS: citrus bacterial spot, ^c^ CBR: crucifer black rot. Red colour in the heat map means presence of the MCP in the strain

**Table 6:** Chemotactic responses toward different concentrations of several apoplastic fluids of *Xanthomonas* pathogenic to citrus and crucifers^a^.

Strain/Concentration (mg mL^−1^)		3.12	6.25	12.5	25	50	100	200
*Xcc* 306 A	Sweet orange	+ ^a^	+	+	+	+	+	+
*Xcc* 62 A		+	+	+	+	+	+	+
*Xcc* 12879 A^w^		+	0	+	+	+	+	+
*Xcc* Iran2 A*		+	+	+	+	+	+	+
*Xec* F1		+	+	+	+	+	+	+
*Xc* 1609		0 ^b^	+	+	+	+	+	+
*Xcc* 306 A	Mexican lime	0	0	0	0	0	0	+
*Xcc* 62 A		+	+	+	+	+	+	+
*Xcc* 12879 A^w^		0	+	+	+	+	+	+
*Xcc* Iran2 A*		+	+	+	+	+	+	+
*Xec* F1		0	0	0	+	+	+	+
*Xc* 1609		+	+	+	+	+	+	+
*Xcc* 306 A	Chinese cabbage	0	+	+	+	+	+	+
*Xcc* 62 A		+	+	+	+	+	+	+
*Xcc* 12879 A^w^		+	+	+	+	+	+	+
*Xcc* Iran2 A*		0	+	+	+	+	+	+
*Xec* F1		0	0	0	+	+	+	+
*Xc* 1609		0	0	+	+	+	+	+

The apoplastic fluid concentration was considered a chemoattractant (+) ^a^ when the average number of bacteria that entered the tip in six replicates from at least two assays was significantly (*p* < 0.05) higher compared to the water control. When *p* > 0.05, no response was considered (0) ^b^.

## Data Availability

Not applicable.
